# Supplementary material for: Novel TiO2/GO-Al2O3 Hollow Fiber Nanofiltration Membrane for Desalination and Lignin Recovery
Source: Membranes (Basel). 2022 Sep 28;12(10):950. doi: 10.3390/membranes12100950 (PMC9608806; doi:10.3390/membranes12100950)
Supplement: Supplementary file 1 [file membranes-12-00950-s001.zip › membranes-1918317-supplementary.pdf]

Supplementary Materials

Novel TiO_2/GO-Al_2O_3 Hollow Fiber Nanofiltration Membrane for Desalination and Lignin Recovery

Xuelong Zhuang[1], Edoardo Magnone[1], Min Chang Shin[1], Jeong In Lee[1], Jae Yeon Hwang[1], Young Chan Choi[2] and Jung Hoon Park[1,*]

[1] Department of Chemical and Biochemical Engineering, Dongguk University, 30, Pildong-ro 1 gil, Jung-gu, Seoul, 04620, South Korea

[2]Fine Dust Research, Korea Institute of Energy Research (KIER), Daejeon, 34129, Korea

* *Correspondence: author.* Tel: +82-2-2260-8598. Fax: +82-2-2260-8729. E-mail: pjhoon@dongguk.edu

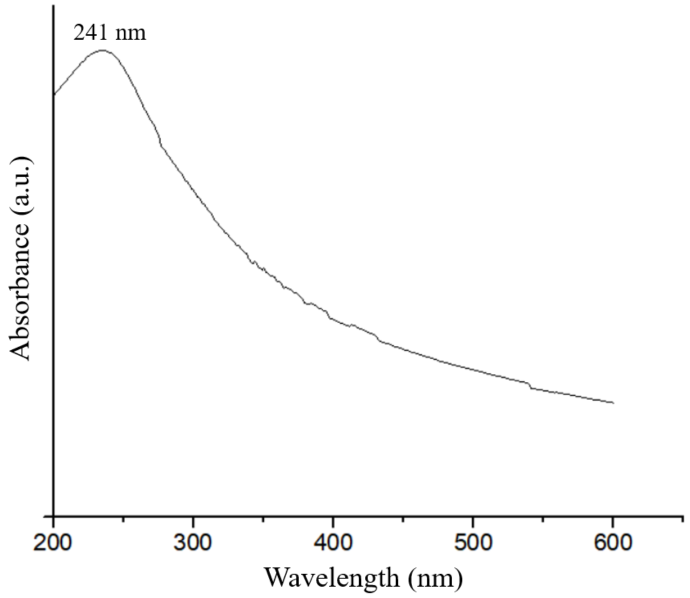

Figure S1. UV-vis spectra of prepared GO.
